# Online bias-aware disease module mining with ROBUST-Web

**DOI:** 10.1093/bioinformatics/btad345

**Published:** 2023-05-26

**Authors:** Suryadipto Sarkar, Marta Lucchetta, Andreas Maier, Mohamed M Abdrabbou, Jan Baumbach, Markus List, Martin H Schaefer, David B Blumenthal

**Affiliations:** Biomedical Network Science Lab, Department of Artificial Intelligence in Biomedical Engineering, Friedrich-Alexander-Universität Erlangen-Nürnberg, Erlangen 91301, Germany; Department of Experimental Oncology, IEO European Institute of Oncology IRCCS, Milan 20139, Italy; Institute for Computational Systems Biology, University of Hamburg, Hamburg 22607, Germany; Biomedical Network Science Lab, Department of Artificial Intelligence in Biomedical Engineering, Friedrich-Alexander-Universität Erlangen-Nürnberg, Erlangen 91301, Germany; Institute for Computational Systems Biology, University of Hamburg, Hamburg 22607, Germany; Chair of Experimental Bioinformatics, TUM School of Life Sciences, Technical University of Munich, Freising 85354, Germany; Department of Experimental Oncology, IEO European Institute of Oncology IRCCS, Milan 20139, Italy; Biomedical Network Science Lab, Department of Artificial Intelligence in Biomedical Engineering, Friedrich-Alexander-Universität Erlangen-Nürnberg, Erlangen 91301, Germany

## Abstract

**Summary:**

We present ROBUST-Web which implements our recently presented ROBUST disease module mining algorithm in a user-friendly web application. ROBUST-Web features seamless downstream disease module exploration via integrated gene set enrichment analysis, tissue expression annotation, and visualization of drug–protein and disease–gene links. Moreover, ROBUST-Web includes bias-aware edge costs for the underlying Steiner tree model as a new algorithmic feature, which allow to correct for study bias in protein–protein interaction networks and further improves the robustness of the computed modules.

**Availability and implementation:**

Web application: https://robust-web.net. Source code of web application and Python package with new bias-aware edge costs: https://github.com/bionetslab/robust-web, https://github.com/bionetslab/robust_bias_aware.

## 1 Introduction

Disease module mining methods (DMMMs), also known as active module identification or *de novo* pathway enrichment methods, discover candidate pathomechanisms in molecular networks based on disease association data obtained from differential gene expression analysis or genome-wide association studies. Among the various recently proposed DMMMs, state-of-the-art methods such as DIAMOnD (Ghiassian *et al.* 2015), DOMINO (Levi *et al.* 2021), and our recently presented tool ROBUST (Bernett *et al.* 2022) rely on a protein–protein interaction (PPI) network and a set of disease-associated seed genes or proteins as input. Here, we present ROBUST-Web, extending ROBUST with two important features:

- A user-friendly web application which allows to run ROBUST in the browser and supports interactive downstream exploration of the computed modules.
- Bias-aware edge costs for the prize-collecting Steiner tree (PCST) model underlying ROBUST, which mitigate a hub node bias of many existing DMMMs (Lazareva *et al.* 2021b), including the original version of ROBUST.

A number of network analysis and visualization tools already exist: For instance, Cytoscape (Shannon *et al.* 2003) and Gephi (Bastian *et al.* 2009) are widely used Java tools for network visualization and exploration. They both require local configuration and/or installation. For Cytoscape, a preliminary version of ROBUST (Sadegh *et al.* 2021) is available as a plugin. There are also some web-based DMMMs, e.g. KeyPathwayMinerWeb (List *et al.* 2016), BiCoN (Lazareva *et al.* 2021a), and the DOMINO web-server (Levi *et al.* 2022). However, unlike ROBUST-Web, none of these tools offers features to link the computed modules to drugs and diseases.

## 2 Web application

**Figure 1:**
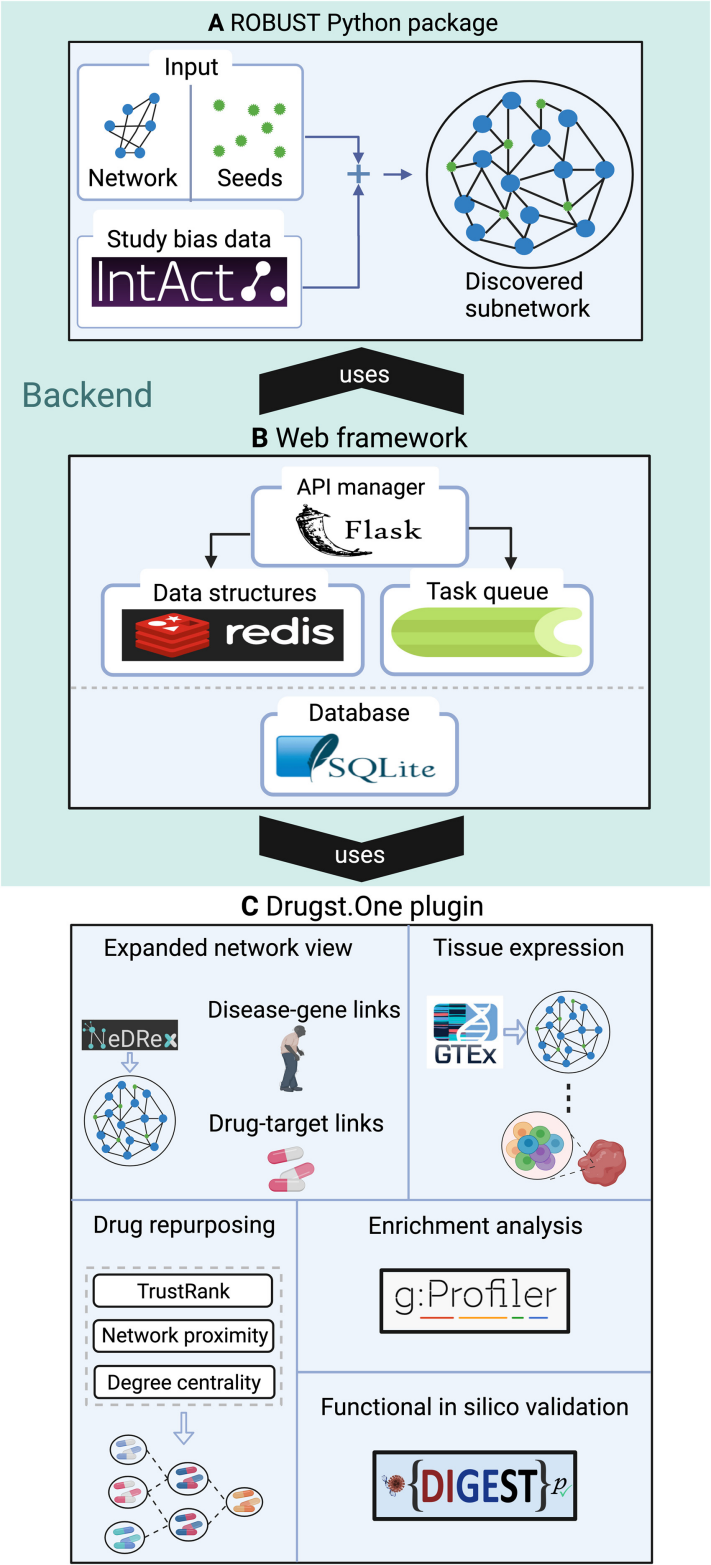
Overview of ROBUST-Web. (A) The ROBUST algorithm is implemented in a Python package. It includes study bias data obtained from IntaAt (see Section 3 for details) and computes a disease module, given a user-provided seed set and PPI network. (B) The web framework calls the Python package and stores the discovered disease module in a database for later retrieval via a stable URL. (C) For result exploration, ROBUST-Web uses the Drugst.One plugin which provides features for tissue expression, drug repurposing, enrichment analysis, and *in silico* validation. Figure generated with BioRender.com.

Figure 1 provides a schematic overview of ROBUST-Web. ROBUST-Web’s most important features are summarized below. Case studies into precocious puberty and COVID-19 which showcase the functionality provided by ROBUST-Web are contained in the supplement.

*Input*. Only a list of seed genes or proteins is required. Users who do not want to provide their own network can select among the STRING (Szklarczyk *et al.* 2019), APID (Alonso-López *et al.* 2019), and BioGRID (Oughtred *et al.* 2019) networks, which are updated monthly via automated downloads from NDEx (Pratt *et al.* 2015).

*Expanded network view*. The disease modules can be enriched with drug–target and disease–gene links obtained from NeDRex (Sadegh *et al.* 2021). NeDRex contains drug–target associations from DrugCentral (Avram *et al.* 2021) and DrugBank (Wishart *et al.* 2018) and disease–gene links from DisGeNET (Piñero *et al.* 2020) and OMIM (Amberger *et al.* 2019). A complete overview of all data sources used for the expanded network view is provided in Supplementary Table S2.

*Enrichment analysis and functional in silico validation of the computed modules*. Supported via queries to the APIs of g: Profiler (Raudvere *et al.* 2019) and DIGEST (Adamowicz *et al.* 2022).

*Tissue expression*. Tissue-specific expression levels for genes contained in the computed modules can be visualized based on data obtained from GTEx (GTEx Consortium 2013).

*Drug repurposing*. Promising drug repurposing candidates targeting the module proteins can be ranked via TrustRank (Gyöngyi *et al.* 2004), network proximity (Guney *et al.* 2016), closeness or degree centrality.

*Implementation*. The backend of ROBUST-Web is written in Python and uses the pcst_fast package (Hegde *et al.* 2014) for Steiner tree computation. The web framework uses Flask as API manager, redis for data structures and cache management, Celery for task queueing, and SQLite for storing the results of user queries. The front-end is written in native HTML, JavaScript, and JQuery, and uses CSS and Bootstrap for styling. Visualization and all functions to support explorative downstream analysis of the computed modules are provided by the Drugst. One plugin (https://drugst.one). ROBUST-Web has been successfully tested on the combinations of browsers and operating systems shown in Table 1.

**Table 1.** Combinations of browsers and operating systems on which ROBUST-Web has been tested.

Operating system	Chrome	Firefox	Edge	Opera	Safari
macOS	✓	✓	✓	✓	✓
Windows	✓	✓	✓	✓	✗
Linux	✓	✓	✗	✓	✗

**Figure 2:**
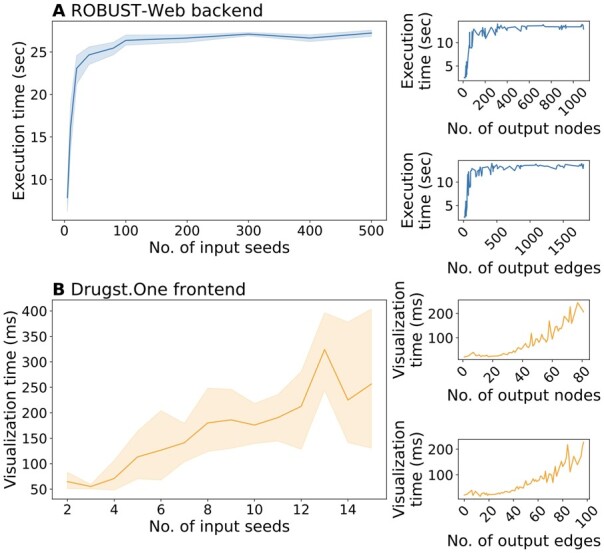
Query times of ROBUST-Web backend (in seconds) (A) and Drugst.One network layouting (in milliseconds) (B) for seed sets, output nodes, and edges of varying sizes.

*Scalability of web application*. To benchmark the scalability of the ROBUST-Web backend, we started with 743 genes related to diabetes mellitus, which we obtained from Feng *et al.* (2022) (we used this gene set because it is large enough to allow subsampling as detailed below). From this gene set, we randomly 10 seed sets of size *k* for each k∈{5,10,20,40,80,100,200,300,400,500}. We then ran ROBUST-Web (bait-usage-based edge costs, BioGRID PPI network) on all of these seed sets and recorded the execution times of the backend. Figure 2A shows the results: Execution times of ROBUST-Web’s backend stabilize when we use more than 200 seeds and disease modules with around 1000 nodes and 1500 edges can be computed and stored in the database in <30 s. Since visualizations of large networks are difficult to interpret, the Drugst.One frontend does not layout disease modules with more than 100 nodes or edges (to visually explore such large disease modules with ROBUST-Web, users can generate views of connected components or 1-hop neighborhoods of selected nodes). To test the scalability of the frontend, we hence subsampled 10 seed sets of size *k* for each k∈{2,3,…,10}, ran ROBUST-Web as for the backend scalability tests, and then measured Drugst.One’s visualization times. The results are shown in Fig. 2B. The Drugst.One plugin takes <0.5 s to layout an output network with 80 nodes and 100 edges.

## 3 Bias-aware edge costs

The ROBUST algorithm is based on a PCST model which, given a graph G=(V,E,c,π) with non-negative edge costs *c* and node prizes π, asks to compute a tree $T=(V_{T},E_{T})\subseteq G$ minimizing $\sum_{uv\in E_{T}}c(uv)+\sum_{u\in V\setminus V_{T}}\pi (u)$. For the original version, we used uniform edge costs c(uv)=1, for all edges *uv*. However, uniform edge costs make high-degree nodes (hubs) very attractive as connectors, which is problematic because hub proteins in PPI networks have been shown to often emerge due to study bias (Schaefer *et al.* 2015). Moreover, existing DMMMs have been shown to inherit this bias by mainly learning from the node degrees instead of the biological knowledge encoded in the edges of PPI networks (Lazareva *et al.* 2021b). To mitigate this problem, we here propose optional bias-aware edge costs


$$c(uv)=\gamma \cdot \max\{f(u),f(v)\}+(1-\gamma )\cdot \frac{\sum_{u^{\prime }v^{\prime }\in E}\max\{f(u^{\prime }),f(v^{\prime })\}}{\left|E\right|},$$

where f(u)≥1 is a score that grows with increasing evidence that PPIs involving protein *u* are over-represented due to study bias (details below) and γ∈[0,1] is a hyper-parameter. If set to γ=1, we fully leverage the information contained in *f*, while setting γ=0 leads to constant edge costs c(uv)=C with $C=\sum_{u^{\prime }v^{\prime }\in E}\max\{f(u^{\prime }),f(v^{\prime })\}/|E|$. Since the optimization problems solved by the ROBUST algorithm are equivalent for all positive constant edge costs, setting γ=0 hence renders the bias-aware costs equivalent to the uniform costs c(uv)=1 used in the original version. We provide three options for the study bias score *f*:

- *Study-attention-based edge costs*: Define f(u) as the number of studies where a protein interaction has been tested that involves *u* (counting both studies where *u* has been tested as bait protein and studies where *u* has been tested as prey protein).
- *Bait-usage-based edge costs*: Restrict to the number of studies where *u* has been tested as a bait protein. Data on study attention and bait usage are obtained from IntAct (Orchard *et al.* 2014) and are updated each month in the web app.
- As a third option, ROBUST can be run with *custom study bias scores* $f(u)\in R_{>0}$.

**Figure 3:**
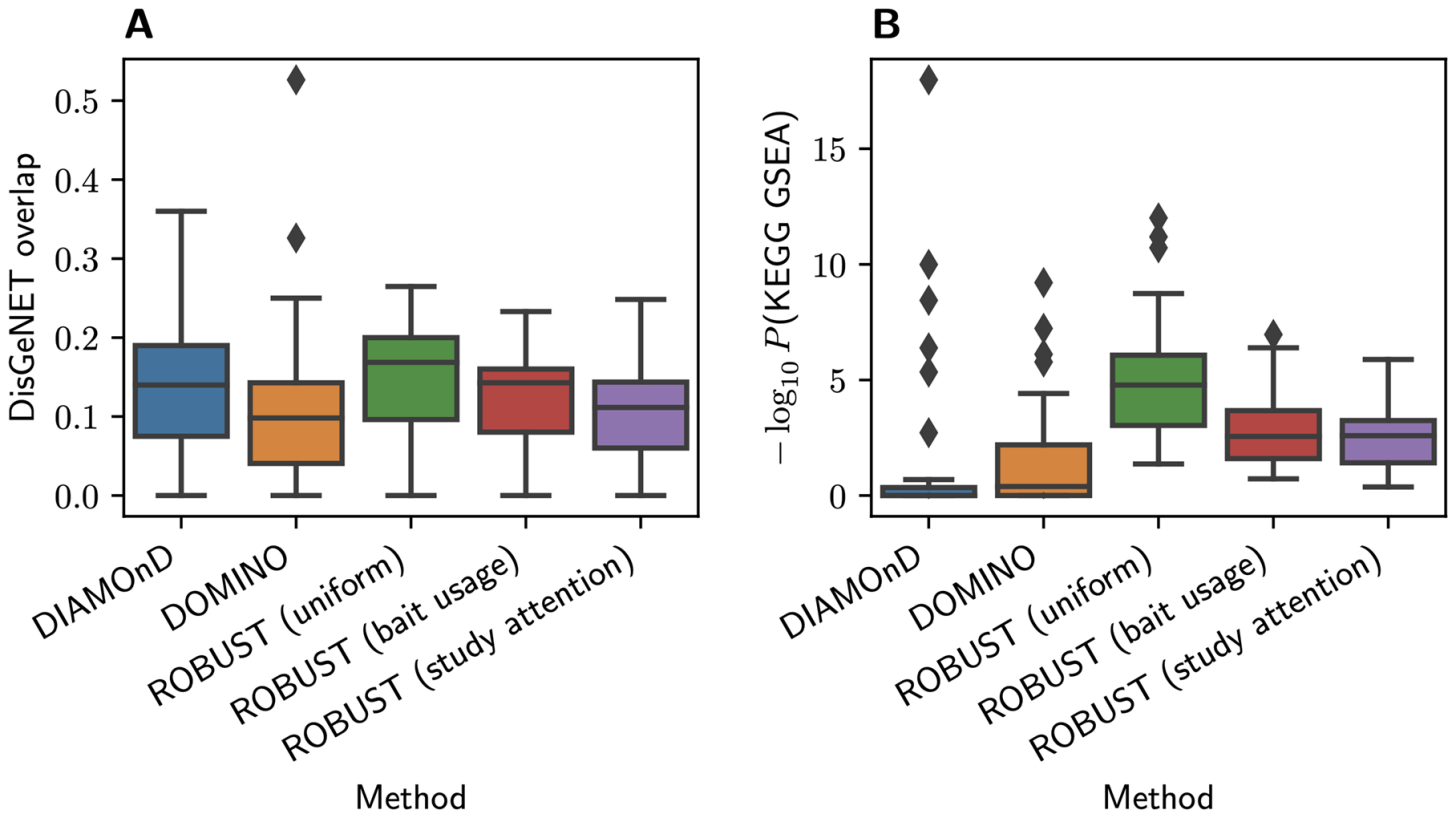
DisGeNET overlap (A) and KEGG gene set enrichment (B) of disease modules computed by different ROBUST versions in comparison to modules computed by DIAMOnD and DOMINO. Bait-usage-based and study-attention-based edge costs were run with γ=1.

*Effect of bias-aware edge costs on functional enrichment of computed disease modules*. We evaluated the effect of the study-attention- and bait-usage-based edge costs by running ROBUST with γ∈{0.0,0.1,…,1.0} and the competitors DIAMOnD and DOMINO, using the protocols suggested by Lazareva *et al.* (2021b). More specifically, we used five publicly available PPI networks—namely APID, BioGRID, HPRD (Keshava Prasad *et al.* 2009), IID (Kotlyar *et al.* 2019), and STRING—along with gene expression data with case/control annotations for Huntington’s disease (HD), Chron’s disease (CD), ulcerative colitis (UC), lung cancer (LC), and amyotrophic lateral sclerosis (ALS). From the gene expression data, we computed condition-specific seed sets by comparing gene expression values for cases and controls via the two-sided Mann–Whitney *U*-test and then marking differentially expressed genes (Bonferroni-adjusted P<0.001) as seeds. Then, we ran all DMMMs on all combinations of seed sets and networks, and quantified functional relevance via (i) gene set enrichment *P*-values of the obtained modules against hand-selected condition-specific KEGG (Kanehisa *et al.* 2016) terms (see Supplementary Table S1) and (ii) overlap coefficients with disease genes obtained from DisGeNET. Note that ROBUST and DOMINO, but not DIAMOnD, sometimes return several disconnected modules. To allow for a uniform evaluation protocol, we computed the evaluation metrics based on their unions. By design, our protocol is hence slightly biased in favor of DIAMOnD. In addition, we carried out case studies into COVID-19 and precocious puberty both with uniform and with bait-usage-based edge costs (see Sections 1 and 2 in the supplement). The results can be summarized as follows:

- Increasing γ indeed decreases the node degrees in the computed modules at the price of reduced functional enrichment (Supplementary Figs S9–S10).
- However, even with γ=1, ROBUST still slightly outperforms the competitors DIAMOnD and DOMINO (Fig. 3).
- Bias-aware edge costs lead to more targeted results containing fewer genes which are extremely richly annotated with a plenitude of not necessarily use-case related terms (Supplementary Fig. S7).

**Figure 4:**
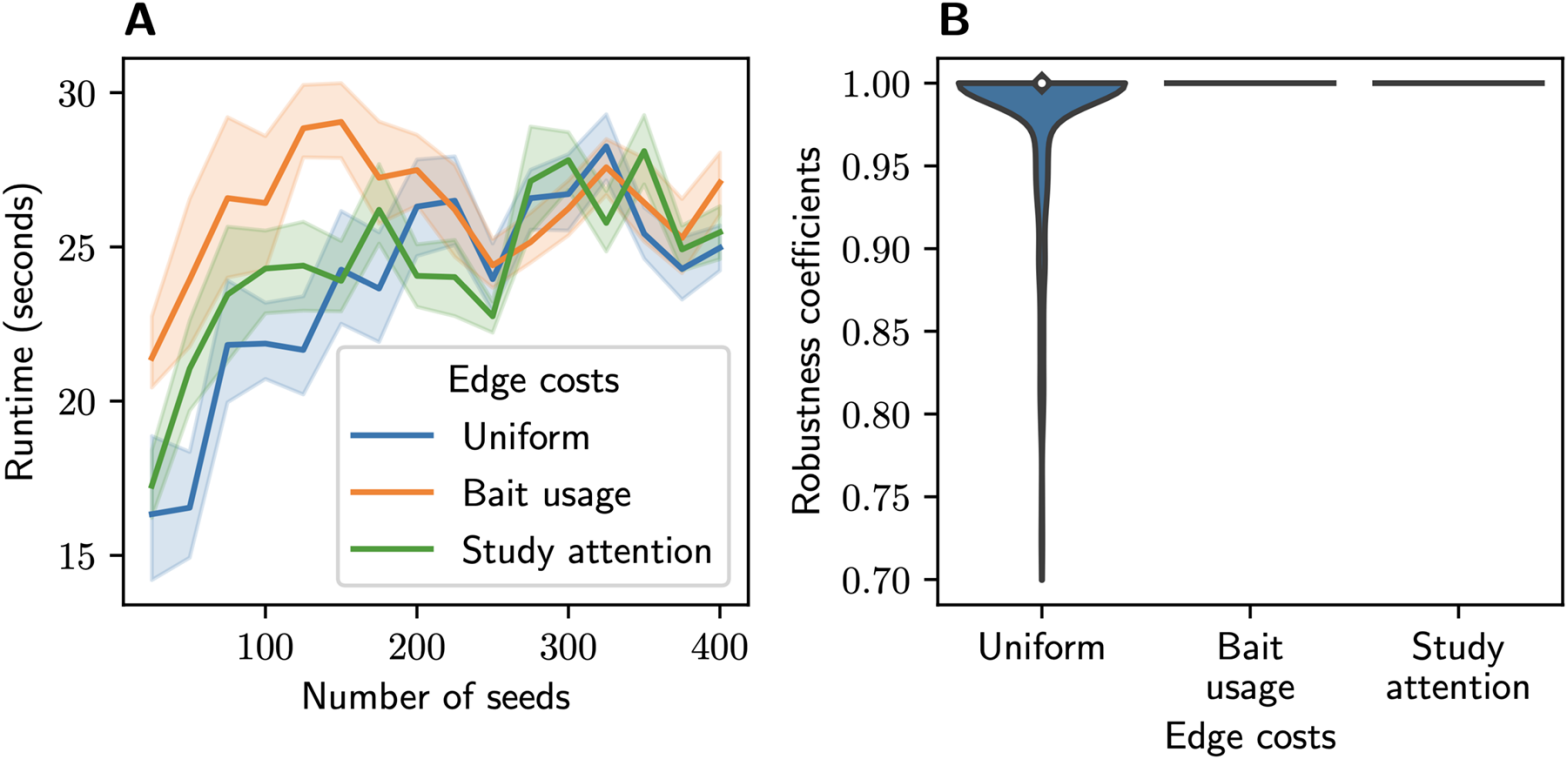
Scalability (A) and robustness (B) of different ROBUST versions. Bait-usage-based and study-attention-based edge costs were run with γ=1.

*Effect of bias-aware edge costs on robustness to random bias and scalability*. The main motivation that led to the development of the original ROBUST algorithm was that existing DMMMs often lack robustness w.r.t. random bias, i.e. yield different disease modules when run several times on equivalent input. To test how the new bias-aware edge costs influence robustness, we used the same protocol as Bernett *et al.* (2022): From IID, OMIM, and DisGeNET, we obtained a human PPI network of experimentally confirmed interactions and seed sets for 929 diseases. For each seed set, we ran ROBUST 20 times with uniform, study-attention-based (γ=1), and bait-usage-based (γ=1) edge costs. Before each of the 20 runs, we shuffled the order in which edges of the PPI network are loaded in the main memory. Then, for each of the three ROBUST configurations, we computed a


$$\text{robustness coefficient}={\left(\begin{array}{c} 20\\ 2 \end{array}\right)}^{-1}\sum_{i=1}^{19}\sum_{j=i+1}^{20}\frac{\left|M_{i}\cap M_{j}\right|}{\left|M_{i}\cup M_{j}\right|}$$

as the mean pairwise Jaccard index of the 20 disease modules $M_{i}$ obtained for the seed set on the 20 randomly re-ordered PPI networks. The robustness coefficient assumes values between 0 and 1 with 1 indicating perfect robustness. In addition, we assessed how bias-aware edge costs affect the runtime of the ROBUST algorithm, by running the three versions of ROBUST on the IID network and randomly sampled seed sets of sizes k∈{25,50,…,400} (10 random seed sets for each *k*). The results are shown in Fig. 4. In terms of runtime, all tested versions of ROBUST performed similarly, but the bias-aware edge costs further improved ROBUST’s robustness: With both the bait-usage-based and the study-attention-based edge costs, ROBUST always computed exactly the same modules when run on randomly shuffled equivalent input.

## Supplementary Material

btad345_Supplementary_Data

## Data Availability

The data underlying this article are available at https://github.com/bionetslab/robust_bias_aware.
