# Correction to: Online bias-aware disease module mining with ROBUST-Web

**DOI:** 10.1093/bioinformatics/btad566

**Published:** 2023-09-14

This is a correction to: Suryadipto Sarkar, Marta Lucchetta, Andreas Maier, Mohamed M Abdrabbou, Jan Baumbach, Markus List, Martin H Schaefer, David B Blumenthal, Online bias-aware disease module mining with ROBUST-Web, *Bioinformatics*, Volume 39, Issue 6, June 2023, btad345, https://doi.org/10.1093/bioinformatics/btad345

The originally published version of this manuscript was incorrectly assigned to vol. 35, issue 6, and has now been correctly placed in volume 39, issue 6. Please note the updated citation information.

This error has been corrected online.

