# Supplementary material for: Online bias-aware disease module mining with ROBUST-Web
Source: Bioinformatics. 2023 May 26;39(6):btad345. doi: 10.1093/bioinformatics/btad345 (PMC10246579; doi:10.1093/bioinformatics/btad345)
Supplement: btad345_Supplementary_Data [file btad345_supplementary_data.pdf]

Online bias-aware disease module mining with ROBUST-Web

Suryadipto Sarkar, Marta Lucchetta, Andreas Maier, Mohamed M. Abdrabbou, Jan Baumbach, Markus List, Martin H. Schaefer, David B. Blumenthal

Supplementary information

1 Case study into comorbidities of severe COVID-19

Owing to the known comorbidities of severe COVID-19 with hypertension, diabetes mellitus, and coronary heart disease, Feng *et al.* (2022) identified eleven candidate genes that seem to play a role in all four conditions (TLR4, NLRP3, MBL2, IL6, IL1RN, IL1B, CX3CR1, CCR5, AGT, ACE, and F2). To elucidate potential mechanisms underlying these comorbidities, we ran ROBUST-Web with the genes identified by Feng *et al.* (2022) as input seeds. We configured ROBUST-Web to use the in-built BioGRID protein-protein interaction (PPI) network (Oughtred *et al.*, 2019), bait-usage-based study bias scores with $\gamma = 1$ (note that this is the default in the web interface), and all other hyper-parameters set to their default values.

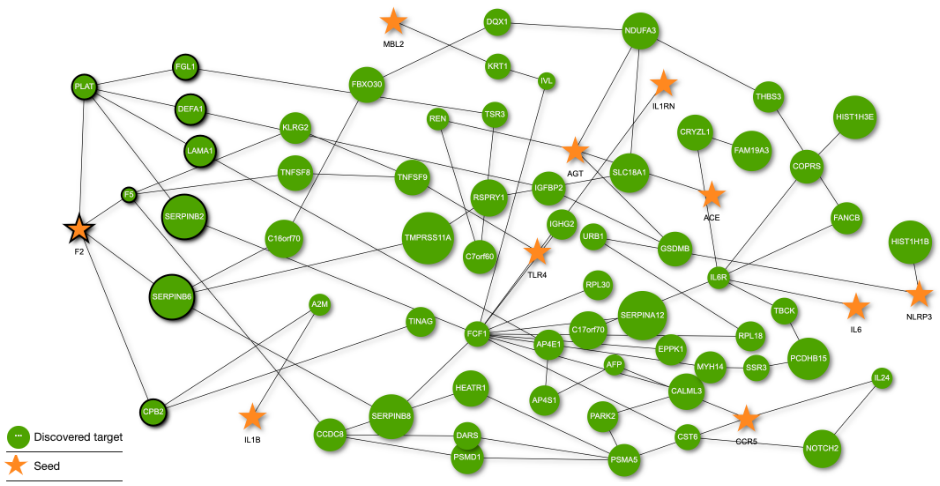

Supplementary Figure 1. COVID-19 disease module computed by ROBUST-Web.

The resulting module is shown in Supplementary Figure 1. In addition to the eleven seeds, it contains 60 newly discovered proteins. Running DIGEST (Adamowicz *et al.*, 2022) on the newly discovered targets to assess the functional coherence of the computed module, we obtained highly significant empirical *P*-values, indicating that the discovered targets might indeed be involved in a joint mechanism (Supplementary Figure 2).

Subsequently, we ran the TrustRank algorithm available via ROBUST-Web's "Drug Search" function to uncover potential drug repurposing candidates targeting the newly discovered proteins. Among the top 20 returned drugs, six drugs target the tissue-type plasminogen activator

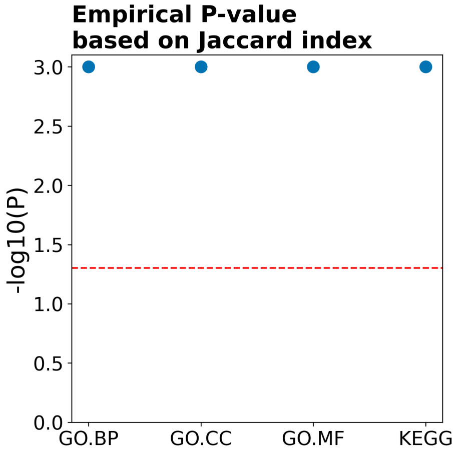

(A) Empirical *P*-values obtained from DIGEST when run on targets discovered by ROBUST-Web.

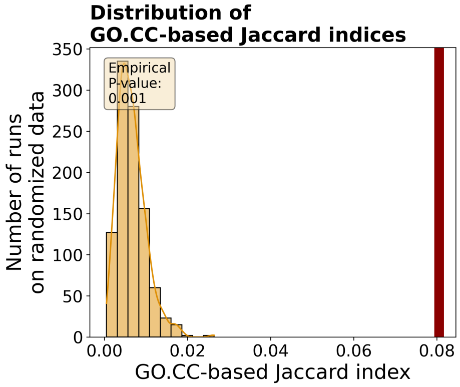

(B) Distribution mean Jaccard indices of GO cellular component annotations of discovered targets (red bar) in comparison to a random background distribution (yellow bars).

Supplementary Figure 2. Results of DIGEST (Adamowicz *et al.*, 2022) validation of functional coherence of the newly discovered targets contained in the COVID-19 disease module shown in Supplementary Figure 1.

(PLAT): ximelagatran, melagatran, dabigatran, dabigatran etexilate, argatroban, and aminocaproic acid. Except for aminocaproic acid, all of these drugs also target prothrombin (F2), which is one of the input seeds.

PLAT is associated with the breakdown of blood clots. Zuo *et al.* (2021) have reported strong correlations between elevated PLAT levels and COVID-19-related hospitalizations, worse respiratory status, mortality and *ex vivo* clotlysis, and spontaneous fibrinolysis. The protein prothrombin encoded by F2 is associated with blood coagulation in humans (Royle *et al.*, 1987; Degen and Davie, 1987). A closer look at the five drugs which target PLAT and F2 further strengthens the link to thrombosis and coagulation: Dabigatran etexilate is an FDA-approved oral thrombin inhibitor administered for the prevention of stroke in patients with atrial fibrillation (Legrand *et al.*, 2011; Connolly *et al.*, 2009). Ximelagatran is an oral thrombin inhibitor mostly used for the prevention of venous thromboembolism after hip or knee replacement (Evans *et al.*, 2004; Eriksson *et al.*, 2002b,a; Heit *et al.*, 2001). Argatroban is a direct thrombin inhibitor used to treat a wide range of thrombotic disorders (McKeage and Plosker, 2001; Dhillon, 2009; Lewis *et al.*, 2003; Yeh and Jang, 2006).

We performed gene set enrichment analysis (GSEA) via ROBUST-Web's inbuilt g:Profiler (Raudvere *et al.*, 2019) interface on the seven neighboring nodes of PLAT and F2 in the computed module (selected nodes with black border in Supplementary Figure 1). Among the top three most significantly enriched terms, two denote pathways related to coagulation (see Supplementary Figure 3). Together, these results suggest that ROBUST-Web can identify potentially actionable coagulation disease mechanisms shared by severe COVID-19 and comorbid disorders.

Results of GO GSEA for COVID-19 disease modules M_b and M_u generated by running ROBUST with, respectively, bait-usage-based ($\gamma = 1$) and uniform edge costs are presented in Supplementary Figure 4, along with GSEA results for their set differences $M_b \setminus M_u$ and $M_u \setminus M_b$. Significantly (adjusted $P < 0.05$) enriched terms were obtained using the GSEApy interface of the Enrichr API (Kuleshov *et al.*, 2016). While there is a rather large overlap between the significantly enriched terms found for M_b and M_u, significantly enriched terms obtained for genes found exclusively with, respectively, bait-usage-based and uniform edge costs are close to disjoint. For instance, the GO Molecular Function term "endopeptidase inhibitor activity" was found only

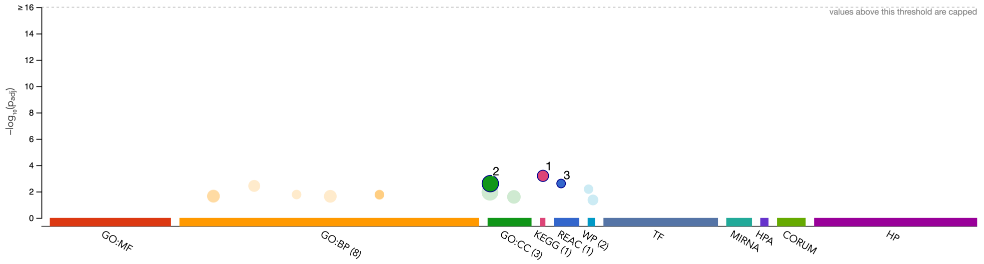

ID	Source	Term ID	Term Name	p_{adj} (query_1)
1	KEGG	KEGG:04610	Complement and coagulation cascades	6.418×10^{-4}
2	GO:CC	GO:0005615	extracellular space	2.497×10^{-3}
3	REAC	REAC:R-HSA-7...	Dissolution of Fibrin Clot	2.458×10^{-3}

**version**	e106_eg53_p16_65fcd97	g:Profiler
**date**	24/10/2022, 15:03:40	
**organism**	hsapiens	

Supplementary Figure 3. GSEA results for the module neighbors of F2 and PLAT (CPB2, SERPINB6, F5, SERPINB2, DEFA1, FGL1, LAMA1; marked nodes in the left part of Supplementary Figure 1) obtained by calling g:Profiler via the ROBUST-Web interface.

with bait-usage-based but not with uniform edge costs. Abdel-Aziz *et al.* (2021) have shown a correlation between high expression of endopeptidases and COVID-19 severity (especially in patients with asthma) and various studies have investigated the use of endopeptidase inhibitors for COVID-19 treatment (Luan *et al.*, 2020; Bojkova *et al.*, 2020; Redondo-Calvo *et al.*, 2022). On the other hand, the top ten GO Molecular Function terms obtained upon performing GSEA on the $M_u \setminus M_b$ genes include very generic terms such as kinase and phosphatase binding.

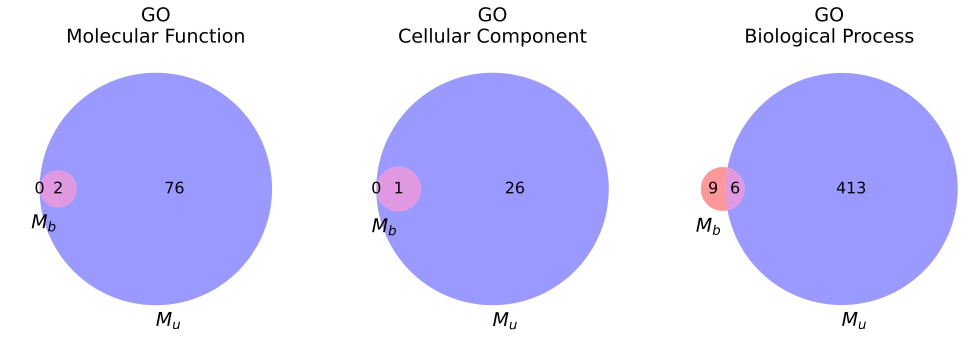

(A)

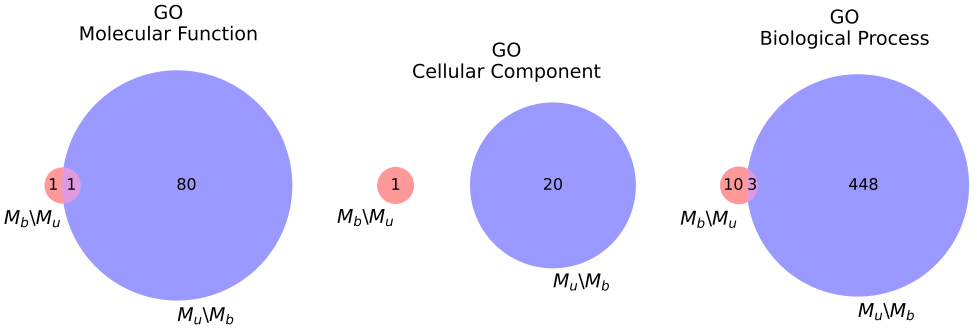

(B)

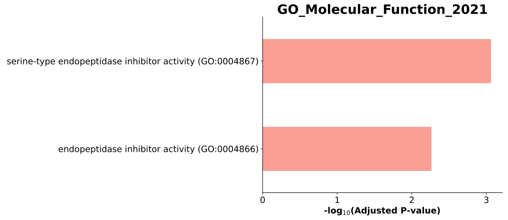

(C)

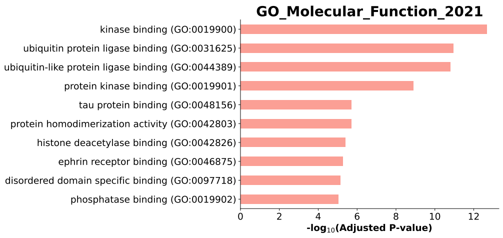

(D)

Supplementary Figure 4. Results of GO GSEA for COVID-19 use case with both uniform and bait usage-based edge costs. (A) Numbers of significanctly enriched GO terms (adjusted P-value below 0.05) for COVID-19 disease modules obtained with bait-usage-based edge costs (M_b) and uniform edge costs (M_u). (B) Significantly enriched GO terms for set differences $M_b \setminus M_u$ and $M_u \setminus M_b$. (C) Significantly enriched GO Molecular Function terms obtained for $M_b \setminus M_u$. (D) Significantly enriched GO Molecular Function terms obtained for $M_u \setminus M_b$.

2 Case study into precocious puberty

Precocious puberty (PP) is a condition where the onset of puberty occurs prematurely in children (before age of 8 in girls and age of 9 in boys, according to Oerter Klein (1999)). The cause of PP is unknown, and treatment is largely symptomatic (Carel and Léger, 2008).

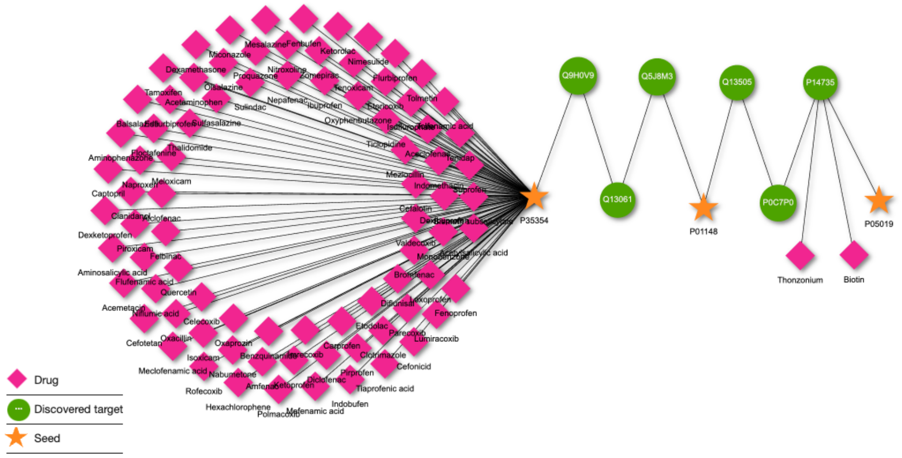

Supplementary Figure 5. Precocious puberty disease module computed by ROBUST-Web together with targeting drugs.

Starting with eight PP-associated proteins obtained from OMIM (Amberger *et al.*, 2019) and DisGeNET (Piñero *et al.*, 2020) (UniProt IDs: P63092, P35354, P01229, P01148, Q5JWF2, P84996, O95467 and P05019), we ran ROBUST-Web using the in-built BioGRID protein-protein interaction (PPI) network (Oughtred *et al.*, 2019), bait-usage-based study bias scores and all hyper-parameters set to their default values. The computed module together with targeting drugs is shown in Supplementary Figure 5. Of the eight input seeds, only three are contained in the computed module, since ROBUST filters seed nodes that are too weakly connected in the PPI network (see Bernett *et al.* (2022) for details). The module contains six newly discovered proteins, including insulin-degrading enzyme (IDE, UniProt ID: P14735). IDE is responsible for the degradation of insulin and natriuretic peptides (Affholter *et al.*, 1990; Ralat *et al.*, 2011) and multiple studies (Chen *et al.*, 2013; Burstein *et al.*, 1987; Sørensen *et al.*, 2012; Hur *et al.*, 2017) have reported elevated levels of both insulin and natriuretic peptides in PP. This leads us to hypothesize that an impairment of IDE might be causally involved in the development of PP.

While most drugs uncovered by ROBUST-Web target the seed protein prostaglandin G/H synthase 2 (UniProt ID: P35354), we find two drugs targeting IDE — biotin and thonzonium. Biotin is a vitamin B7 supplement obtained naturally from different dietary sources. Biotin starvation, although rare, is connected to several clinical conditions including insulin pathway impairment (Salvador-Adriano *et al.*, 2014). Thonzonium is known to play a vital role in bone anti-resorption (Zhu *et al.*, 2016) and Hur *et al.* (2017) have reported a decline in bone mineral density and bone strength and an increase in bone resorption markers in PP. This suggests that both biotin and thonzonium are potential drug repurposing candidates in PP, which encourages further investigation into their effects on insulin homeostasis through IDE control.

Subsequently, we performed enrichment analysis on the six newly discovered targets, using ROBUST-Web's in-built g:Profiler interface (Raudvere *et al.*, 2019). The analysis returned endoplasmic reticulum (ER) organization as the only significantly enriched term (see Supplementary Figure 6). In mice, Linz *et al.* (2015) have linked ER stress during puberty to impaired bone development, which further corroborates the functional relevance of the disease module computed

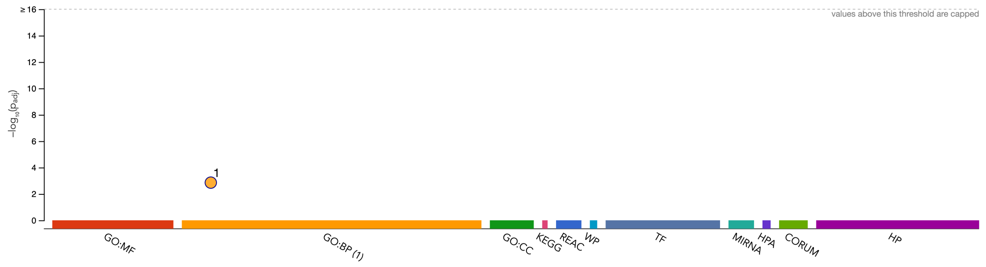

ID	Source	Term ID	Term Name	p_{adj} (query_1)
1	GO:BP	GO:0007029	endoplasmic reticulum organization	1.398×10^{-3}

**version**	e106_eg53_p16_65fcd97
**date**	24/10/2022, 08:48:44
**organism**	hsapiens

g:Profiler

Supplementary Figure 6. GSEA results for the discovered PP targets shown in Supplementary Figure 5 obtained by calling g:Profiler via the ROBUST-Web interface.

by ROBUST-Web.

Results of GO GSEA for PP disease modules M_b and M_u generated by running ROBUST with, respectively, bait-usage-based ($\gamma = 1$) and uniform edge costs are presented in Supplementary Figure 7, along with GSEA results for their set differences $M_b \setminus M_u$ and $M_u \setminus M_b$. Both M_b and M_u were computed as for the COVID-19 use case and significantly enriched terms were again obtained using GSEApy. Again, significantly enriched terms for M_u and M_b overlap significantly, whereas significantly enriched terms for $M_b \setminus M_u$ and $M_u \setminus M_b$ are close to disjoint. An interesting example of a term that was found only with bait-usage-based edge costs is GO Cellular Component term “mitochondrial intermembrane space bridging (MIB) complex”. MIB-1, which is part of the MIB complex, is one of the main markers of cell proliferation (Spyratos *et al.*, 2002; Querzoli *et al.*, 1996; Tortori-Donati *et al.*, 1999; Ramsay *et al.*, 1995; Scalzo *et al.*, 1998; Diebold *et al.*, 2017), which is a critical component of puberty (particularly relating to testicular growth in males and breast development in females) (Naccarato *et al.*, 2000; Koskenniemi *et al.*, 2017; Marshall and Plant, 1996). On the other hand, the top ten most significantly enriched GO Celular Component terms for $M_u \setminus M_b$ genes include very generic terms such as such as “transferase complex, transferring phosphorus-containing groups”, “axon”, “intracellular membrane-bounded organelle”, “protein kinase complex”, “nuclear envelope lumen”, and “endoplasmic reticulum lumen”. That is, genes obtained with uniform edge costs only lead to very generic enrichment results.

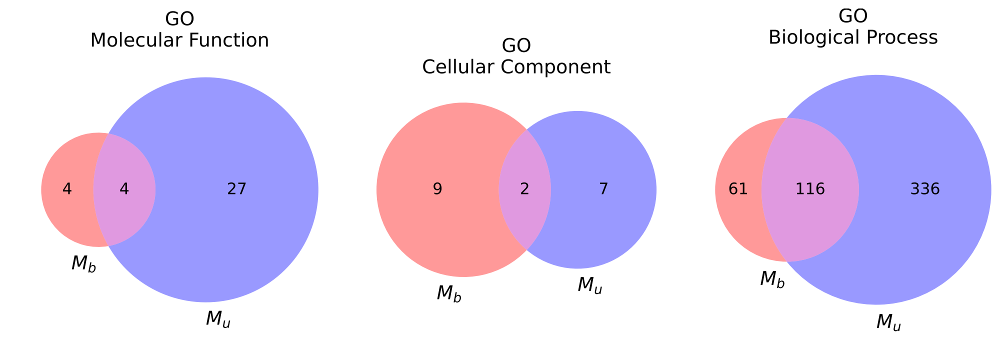

(A)

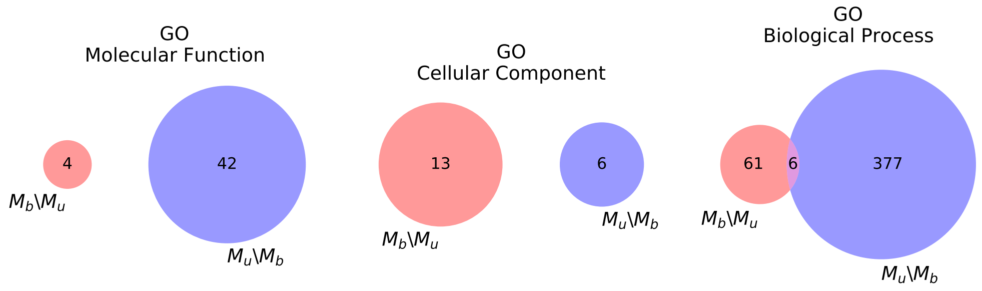

(B)

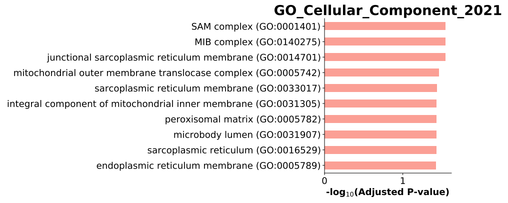

(C)

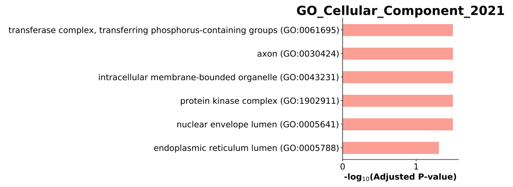

(D)

Supplementary Figure 7. Results of GO GSEA for PP use case with both uniform and bait usage-based edge costs. (A) Numbers of significanctly enriched GO terms (adjusted *P*-value below 0.05) for PP disease modules obtained with bait-usage-based edge costs (M_b) and uniform edge costs (M_u). (B) Significantly enriched GO terms for set differences $M_b \setminus M_u$ and $M_u \setminus M_b$. (C) Significantly enriched GO Molecular Function terms obtained for $M_b \setminus M_u$. (D) Significantly enriched GO Molecular Function terms obtained for $M_u \setminus M_b$.

3 Further supplementary information

Association between bait usage scores and functional enrichment. In both the COVID-19 and the PP case study (see Supplementary Figures 4 and 7), we obtained much fewer significantly enriched GO terms when running ROBUST with bait-usage-based rather than with uniform edge costs. A likely explanation for this is that genes with large bait usage scores are over-represented in gene annotation databases, as suggested by Haynes *et al.* (2018). To assess the plausibility of this explanation, we collected the top 20 genes with the largest bait usage scores $f(u)$ and the bottom 20 genes with the smallest scores bait usage $f(u)$. From each of the two sets, we then subsampled 100 random subsets of size 10 and carried out GO GSEA for all of them. Distributions of the numbers of obtained significantly enriched terms are shown in Supplementary Figure 8. Indeed, for all three GO annotation types, significantly more enriched terms are obtained for the subsets of the top 20 genes than for the subsets of the bottom 20 genes.

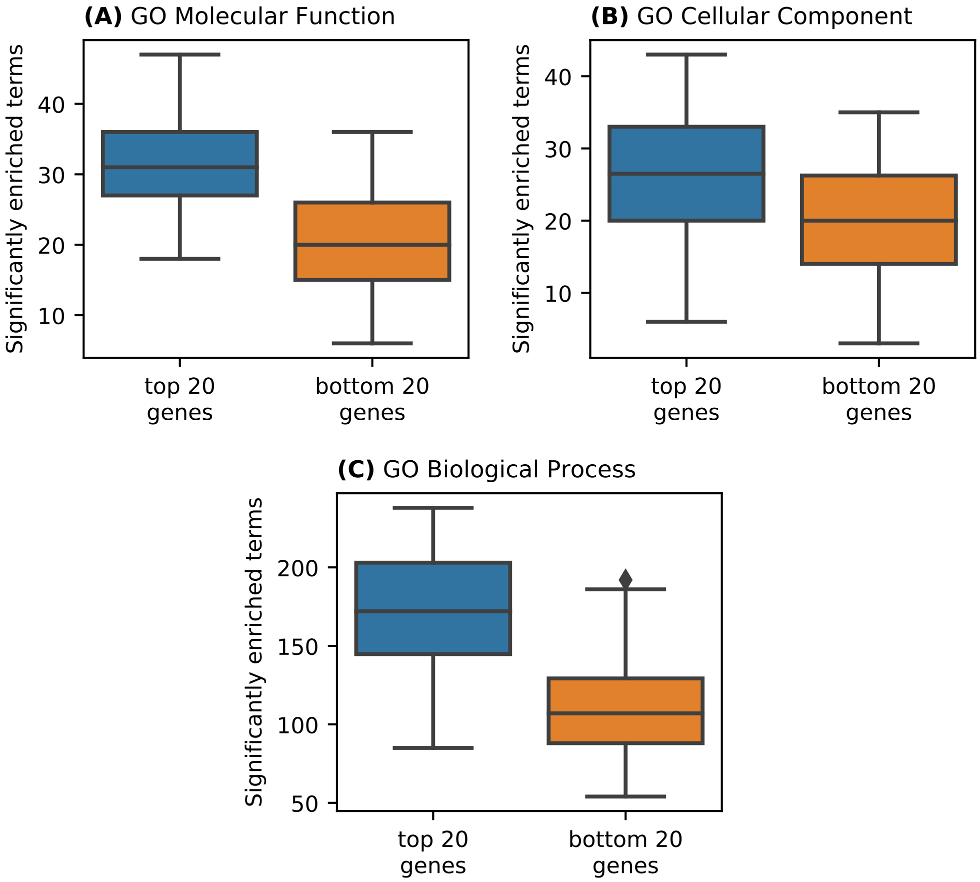

Supplementary Figure 8. Distributions of significantly enriched GO terms obtained for 100 random size-10 subsets of the top 20 genes with the largest bait usage scores and the bottom 20 genes with the smalles scores. (A) Numbers of GO Molecular Function terms. (B) Numbers of GO Cellular Component terms. (C) Numbers of GO Biological Process terms.

Supplementary Table 1. Details on data used for functional relevance validation. Gene expression data was obtained from Gene Expression Omnibus (GEO) (Barrett *et al.*, 2013), using the GEO2R R interface (https://www.ncbi.nlm.nih.gov/geo/geo2r/). DisGeNET (v7.0) associations were obtained using nDEx (Pratt *et al.*, 2015). The KEGG pathways were obtained from the KEGG DISEASE Database (https://www.genome.jp/kegg/disease/).

Disease	Accession code	KEGG pathway	DisGeNET identifier
Huntington's disease	GSE3790	hsa05016	C0020179
Chron's disease	GSE75214	hsa04621	C0021390
		hsa04060	
		hsa04630	
		hsa05321	
		hsa04140	
Ulcerative colitis	GSE75214	hsa04060	C0009324
		hsa04630	
		hsa05321	
Lung cancer	GSE30219	hsa05223	C1737250
Amyotrophic lateral sclerosis	GSE112680	hsa05014	C0002736

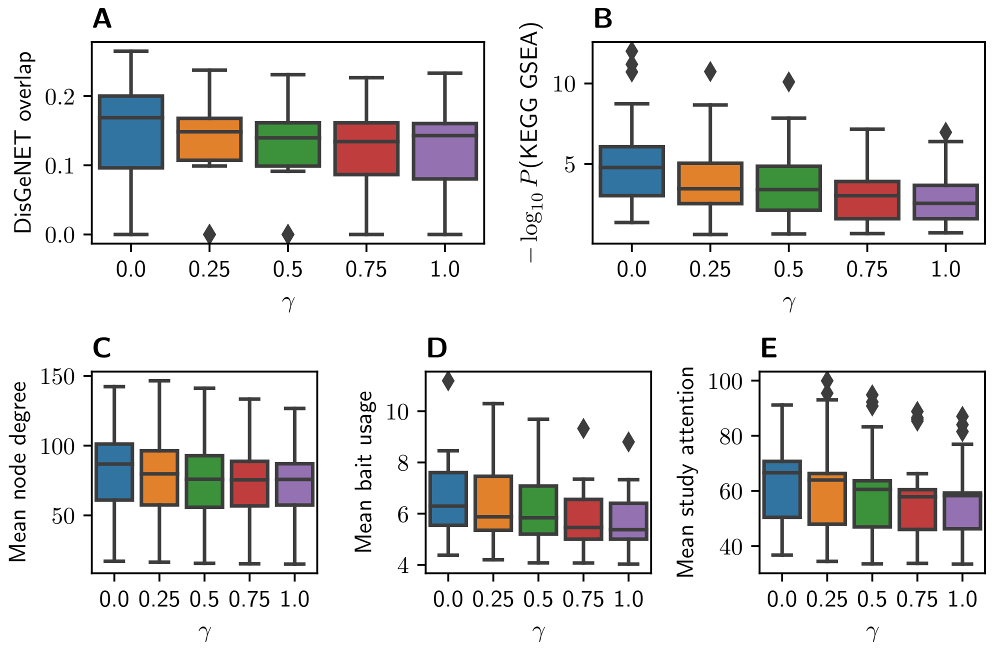

Supplementary Figure 9. Effect of varying γ with bait-usage-based edge costs on DisGeNET overlap (A), KEGG GSEA P-values (B), mean node degree (C), mean bait usage (D), and mean study attention (E) of proteins contained in the returned modules. Analyses were carried out using the gene expression datasets, KEGG pathways, and DisGeNET identifiers detailed in Supplementary Table 1.

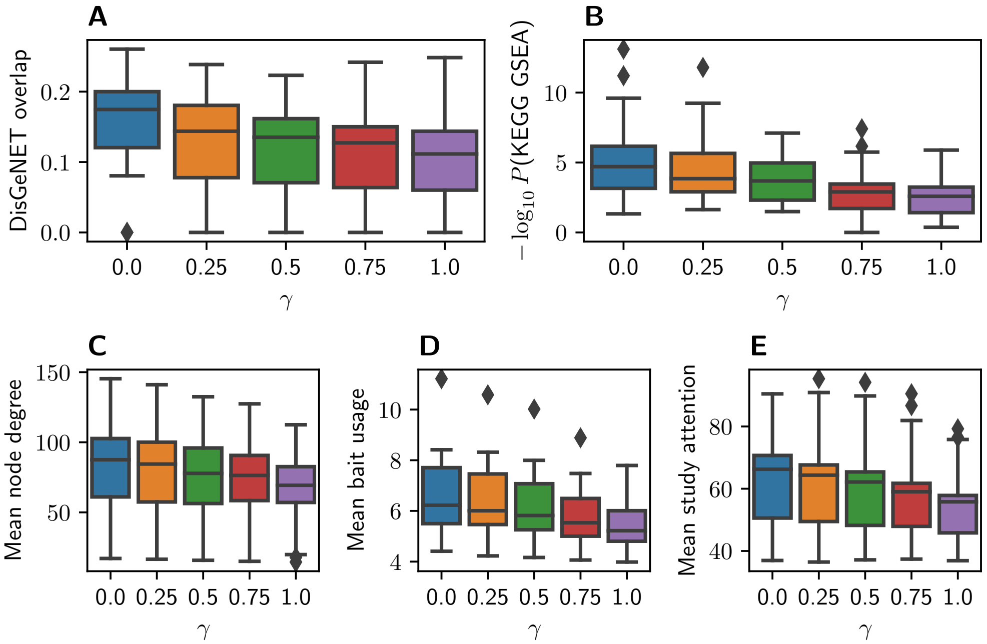

Supplementary Figure 10. Effect of varying γ with study-attention-based edge costs on DisGeNET overlap (A), KEGG GSEA (B), mean node degree (C), mean bait usage (D), and mean study attention (E) of proteins contained in the returned modules. Analyses were carried out using the gene expression datasets, KEGG pathways, and DisGeNET identifiers detailed in Supplementary Table 1.

Supplementary Table 2. Databases queried by the Drugst.One plugin used for result exploration in ROBUST-Web.

Database	Version	Association type
ChEMBL	27	protein-drug
DGIdb	4.2.0	protein-drug
DrugCentral	Feb 2023	protein-drug & drug-disease
DisGeNET	Feb 2023	protein-disease
CTD	Feb 2023	drug-disease
DrugBank	Feb 2023	protein-drug & drug-disease
OMIM (via NeDRex)	Dec 2022	protein-disease

References

Abdel-Aziz, M. I. *et al.* (2021). Association of endopeptidases, involved in SARS-CoV-2 infection, with microbial aggravation in sputum of severe asthma. *Allergy*, **76**(6), 1917–1921.

Adamowicz, K. *et al.* (2022). Online in silico validation of disease and gene sets, clusterings, or subnetworks with DIGEST. *Brief. Bioinform.*, **23**(4), bbac247.

Affholter, J. A. *et al.* (1990). Insulin-degrading enzyme: stable expression of the human comple-

mentary dna, characterization of its protein product, and chromosomal mapping of the human and mouse genes. *Mol. Endocrinol.*, **4**(8), 1125–1135.

Amberger, J. S. *et al.* (2019). OMIM.org: leveraging knowledge across phenotype-gene relationships. *Nucleic Acids Res.*, **47**(D1), D1038–D1043.

Barrett, T. *et al.* (2013). NCBI GEO: archive for functional genomics data sets–update. *Nucleic Acids Res.*, **41**(Database issue), D991–D995.

Bernett, J. *et al.* (2022). Robust disease module mining via enumeration of diverse prize-collecting Steiner trees. *Bioinformatics*, **38**(6), 1600–1606.

Bojkova, D. *et al.* (2020). Aprotinin inhibits SARS-CoV-2 replication. *Cells*, **9**(11).

Burstein, S. *et al.* (1987). Elevated insulin levels in precocious puberty (PP). *Pediatr. Res.*, **21**(4), 173.

Carel, J.-C. and Léger, J. (2008). Precocious puberty. *N. Engl. J. Med.*, **358**(22), 2366–2377.

Chen, Q.-l. *et al.* (2013). Serum aminoterminal proctype natriuretic peptide in girls with idiopathic central precocious puberty during GNRHA treatment. *Int. J. Pediatr. Endocrinol.*, (1), 1.

Connolly, S. J. *et al.* (2009). Dabigatran versus warfarin in patients with atrial fibrillation. *N. Engl. J. Med.*, **361**(12), 1139–1151.

Degen, S. J. F. and Davie, E. W. (1987). Nucleotide sequence of the gene for human prothrombin. *Biochemistry*, **26**(19), 6165–6177.

Dhillon, S. (2009). Argatroban. *Am. J. Cardiovasc. Drugs*, **9**(4), 261–282.

Diebold, M. *et al.* (2017). Prognostic value of mib-1 proliferation index in solitary fibrous tumors of the pleura implemented in a new score–a multicenter study. *Respir. Res.*, **18**(1), 1–8.

Eriksson, B. I. *et al.* (2002a). A dose-ranging study of the oral direct thrombin inhibitor, ximelagatran, and its subcutaneous form, melagatran, compared with dalteparin in the prophylaxis of thromboembolism after hip or knee replacement: Methro i. *Thromb. Haemost.*, **87**(02), 231–237.

Eriksson, B. I. *et al.* (2002b). Ximelagatran and melagatran compared with dalteparin for prevention of venous thromboembolism after total hip or knee replacement: the METHRO II randomised trial. *Lancet*, **360**(9344), 1441–1447.

Evans, H. C. *et al.* (2004). Ximelagatran/melagatran. *Drugs*, **64**(6), 649–678.

Feng, S. *et al.* (2022). Potential genes associated with COVID-19 and comorbidity. *Int. J. Med. Sci.*, **19**(2), 402.

Haynes, W. A. *et al.* (2018). Gene annotation bias impedes biomedical research. *Sci. Rep.*, **8**(1), 1362.

Heit, J. A. *et al.* (2001). Comparison of the oral direct thrombin inhibitor ximelagatran with enoxaparin as prophylaxis against venous thromboembolism after total knee replacement: a phase 2 dose-finding study. *Arch. Intern. Med.*, **161**(18), 2215–2221.

Hur, J. H. *et al.* (2017). Insulin resistance and bone age advancement in girls with central precocious puberty. *Ann. Pediatr. Endocrinol. Metab.*, **22**(3), 176.

Koskenniemi, J. J. *et al.* (2017). Testicular growth and development in puberty. *Curr. Opin. Endocrinol. Diabetes Obes.*, **24**(3), 215–224.

Kuleshov, M. V. *et al.* (2016). Enrichr: a comprehensive gene set enrichment analysis web server 2016 update. *Nucleic Acids Res.*, **44**(W1), W90–W97.

Legrand, M. *et al.* (2011). The use of dabigatran in elderly patients. *Arch. Intern. Med.*, **171**(14), 1285–1286.

Lewis, B. E. *et al.* (2003). Argatroban anticoagulation in patients with heparin-induced thrombocytopenia. *Arch. Intern. Med.*, **163**(15), 1849–1856.

Linz, A. *et al.* (2015). ER stress during the pubertal growth spurt results in impaired long-bone growth in chondrocyte-specific ERp57 knockout mice. *J. Bone Miner. Res.*, **30**(8), 1481–1493.

Luan, B. *et al.* (2020). Targeting proteases for treating COVID-19. *J. Proteome Res.*, **19**(11), 4316–4326.

Marshall, G. and Plant, T. (1996). Puberty occurring either spontaneously or induced precociously in rhesus monkey (macaca mulatta) is associated with a marked proliferation of sertoli cells. *Biol. Reprod.*, **54**(6), 1192–1199.

McKeage, K. and Plosker, G. L. (2001). Argatroban. *Drugs*, **61**(4), 515–522.

Naccarato, A. G. *et al.* (2000). Bio-morphological events in the development of the human female mammary gland from fetal age to puberty. *Virchows Arch.*, **436**, 431–438.

Oerter Klein, K. (1999). Precocious puberty: who has it? who should be treated? *J. Clin. Endocrinol. Metab.*, **84**(2), 411–414.

Oughtred, R. *et al.* (2019). The BioGRID interaction database: 2019 update. *Nucleic Acids Res.*, **47**(D1), D529–D541.

Piñero, J. *et al.* (2020). The DisGeNET knowledge platform for disease genomics: 2019 update. *Nucleic Acids Res.*, **48**(D1), D845–D855.

Pratt, D. *et al.* (2015). NDEx, the network data exchange. *Cell Syst.*, **1**(4), 302–305.

Querzoli, P. *et al.* (1996). MIB-1 proliferative activity in invasive breast cancer measured by image analysis. *J. Clin. Pathol.*, **49**(11), 926–930.

Ralat, L. A. *et al.* (2011). Insulin-degrading enzyme modulates the natriuretic peptide-mediated signaling response. *J. Biol. Chem.*, **286**(6), 4670–4679.

Ramsay, J. A. *et al.* (1995). Mib-1 proliferative activity is a significant prognostic factor in primary thick cutaneous melanomas. *J. Invest. Dermatol.*, **105**(1), 22–26.

Raudvere, U. *et al.* (2019). g:Profiler: a web server for functional enrichment analysis and conversions of gene lists (2019 update). *Nucleic Acids Res.*, **47**(W1), W191–W198.

Redondo-Calvo, F. J. *et al.* (2022). Aprotinin treatment against SARS-CoV-2: A randomized phase III study to evaluate the safety and efficacy of a pan-protease inhibitor for moderate COVID-19. *Eur. J. Clin. Invest.*, **52**(6), e13776.

Royle, N. *et al.* (1987). Human genes encoding prothrombin and ceruloplasmin map to 11p11–q12 and 3q21–24, respectively. *Somat. Cell Mol. Genet.*, **13**(3), 285–292.

Salvador-Adriano, A. *et al.* (2014). Insulin sensitivity is inversely related to cellular energy status, as revealed by biotin deprivation. *Am. J. Physiol. Endocrinol. Metab.*, **306**(12), E1442–E1448.

Scalzo, D. A. *et al.* (1998). Cell proliferation rate by mib-1 immunohistochemistry predicts postradiation recurrence in prostatic adenocarcinomas. *Am. J. Clin. Pathol.*, **109**(2), 163–168.

Sørensen, K. *et al.* (2012). Serum IGF1 and insulin levels in girls with normal and precocious puberty. *Eur. J. Endocrinol.*, **166**(5), 903–910.

Spyratos, F. *et al.* (2002). Correlation between MIB-1 and other proliferation markers: clinical implications of the MIB-1 cutoff value. *Cancer*, **94**(8), 2151–2159.

Tortori-Donati, P. *et al.* (1999). Extraventricular neurocytoma with ganglionic differentiation associated with complex partial seizures. *AJNR Am. J. Neuroradiol.*, **20**(4), 724–727.

Yeh, R. W. and Jang, I.-K. (2006). Argatroban: update. *Am. Heart J.*, **151**(6), 1131–1138.

Zhu, X. *et al.* (2016). Thonzonium bromide inhibits RANKL-induced osteoclast formation and bone resorption in vitro and prevents LPS-induced bone loss in vivo. *Biochem. Pharmacol.*, **104**, 118–130.

Zuo, Y. *et al.* (2021). Plasma tissue plasminogen activator and plasminogen activator inhibitor-1 in hospitalized COVID-19 patients. *Sci. Rep.*, **11**(1), 1–9.
